# ‘Paging Podiatry!’: an audit of acute inpatient podiatry referrals

**DOI:** 10.1186/1757-1146-4-S1-P13

**Published:** 2011-05-20

**Authors:** Sarah Dallimore, Katrina Richards

**Affiliations:** 1 Eastern Health Podiatry Department, Australia

## Background

In 2010, the Box Hill Hospital podiatry department increased staffing levels from 0.6 to 1.6 EFT. Historically a limited High Risk Foot outpatient service, this increase meant there was more time available to build the much needed inpatient service. Promotion of the new service has resulted in a substantial increase in podiatry referrals from the acute wards. The Podiatry Department has also established close links with the Vascular Surgery Department and is a part of their Outpatient Clinic. Once received, the podiatry referrals are routinely triaged into prioritisation categories, according to the severity of the described complaint and any reported co-morbidities. Category 1 and 2 referrals essentially revolve around active foot wounds and complications that require an immediate podiatry response. Category 3 and 4 referrals are less medically urgent and not seen as a priority to the service.

## Method

All inpatient referrals received from January until October 2010 were recorded on an excel spreadsheet. Included for analysis was referring unit and ward, and the triaged prioritisation category.

## Results

A total of 263 referrals were received in this period. The greatest number of referrals came from the Medical and Vascular Units: General Medical B 16.7%; General Medical A 14.8%; Rapid Assessment Medical Unit (RAMU) 14.4%; and Vascular 14.1%.Of these four top referring units, the Vascular Unit had the greatest number of appropriate referrals with 91.9% being either a Category 1 or 2. General Medical A had 44.74%, General Medical B 52.27% and RAMU 47.37%.

## Conclusion

The Vascular Unit had the most number of Category 1 and 2 referrals. This is most likely due to the increased number of patients with high-risk feet under this unit and their understanding of podiatry’s role with the High Risk Foot. The Medical Units referred the most to podiatry, however approximately half of the referrals received were classified as non-urgent and not required to be seen by the podiatrist if time did not permit.

